# Neoadjuvant chemoimmunotherapy cycle number selection for non-small cell lung cancer and clinical outcomes: a real-world analysis

**DOI:** 10.3389/fonc.2023.1200625

**Published:** 2023-09-05

**Authors:** Baihua Zhang, Xiaotong Guo, Ran Jia, Zhan Wang, Jie Wu, Xiaoyan Chen, Jigang Li, Desong Yang, Xu Li, Wenxiang Wang, Qin Xiao

**Affiliations:** ^1^Department of Thoracic Surgery, National Cancer Center/National Clinical Research Center for Cancer/Cancer Hospital and Shenzhen Hospital, Chinese Academy of Medical Sciences and Peking Union Medical College, Shenzhen, China; ^2^Department of Thoracic Surgery, Hunan Clinical Medical Research Center of Accurate Diagnosis and Treatment for Esophageal Carcinoma, Hunan Cancer Hospital and The Affiliated Cancer Hospital of Xiangya School of Medicine, Central South University, Changsha, Hunan, China; ^3^Department of Medical Oncology, Lung Cancer and Gastrointestinal Unit, Hunan Cancer Hospital and The Affiliated Cancer Hospital of Xiangya School of Medicine, Central South University, Changsha, Hunan, China; ^4^Department of Pathology, Hunan Cancer Hospital and The Affiliated Cancer Hospital of Xiangya School of Medicine, Central South University, Changsha, Hunan, China; ^5^Department of Thoracic Radiation Oncology, National Cancer Center/National Clinical Research Center for Cancer/Cancer Hospital and Shenzhen Hospital, Chinese Academy of Medical Sciences and Peking Union Medical College, Changsha, China; ^6^Key Laboratory of Translational Radiation Oncology, The First Department of Thoracic Radiation Oncology, Hunan Cancer Hospital, The Affiliated Cancer Hospital of Xiangya School of Medicine, Central South University, Changsha, Hunan, China

**Keywords:** non-small cell lung cancer, neoadjuvant chemoimmunotherapy, treatment cycles, adverse events, morbidity

## Abstract

**Objectives:**

Neoadjuvant chemoimmunotherapy is the optimal choice in the treatment of NSCLC; however, the optimal number of therapeutic cycles remains unclear. The primary aim of this study was to determine the optimal number of neoadjuvant therapeutic cycles in NSCLC.

**Methods:**

This study was a real-world clinical analysis that included patients who received neoadjuvant chemoimmunotherapy followed by surgery from January 2020 to August 2022. Patients were divided into two groups based on the number of therapeutic cycles: 2-cycle group and 3-4-cycles group. The primary endpoint was the major pathological response (MPR) rate.

**Results:**

A total of 251 patients were included: 150 in the 2-cycle group and 101 in the 3-4-cycles group. Baseline characteristics were well-balanced between the groups. The MPR in the 2-cycle group was 57.3% and not significantly different from that of 57.4% in the 3-4-cycles group (p=0.529). Thirty-two patients (31.7%) in the 3-4-cycles group underwent surgery > 42 days after the final cycle of neoadjuvant therapy, significantly more than the 24 patients (16.0%) in the 2-cycle group (p=0.003). The incidence of adverse events related to neoadjuvant therapy was higher in the 3-4-cycles vs 2-cycle groups (72.3% versus 58.0%, respectively; p=0.021), while the 2-cycle group had a higher rate of postoperative morbidities (28.0% versus 12.9%, respectively; p=0.004). Additionally, for patients with ≤ 44.2% regression in diameter on computed tomography after two cycles of treatment, the MPR rate was higher in the 3-4-cycles vs 2-cycle group (47.3% versus 29.9%, respectively; p=0.048). For cases with programmed death-ligand 1 expression, regarding tumor proportion score ≤ 10%, 3-4 cycles of neoadjuvant treatment increased the MPR rate compared with 2 cycles (37.5% versus 9.5%, respectively; p=0.041).

**Conclusion:**

Our data support the positive role of chemoimmunotherapy in the neoadjuvant treatment of NSCLC. Extending to 3–4 cycles instead of 2 cycles of neoadjuvant chemoimmunotherapy may improve the safety of surgery and result in a lower incidence of postoperative morbidities; however, the MPR rate may not increase significantly. CT re-evaluation during treatment and PD-L1 expression at initial diagnosis are potential indicators to guide the choice of the number of therapeutic cycles.

## Introduction

With the development of immune checkpoint inhibitors, including programmed cell death-ligand 1 (PD-L1) and programmed cell death protein-1 (PD-1) inhibitors, the systemic therapy of non-small cell lung cancer (NSCLC) has entered the era of immunotherapy. Recently, increasing numbers of prospective phase II/III clinical trials have reported positive results with neoadjuvant chemotherapy combined with PD-1/PD-L1 inhibitors compared with chemotherapy or immunotherapy alone in resectable NSCLC (1–5). Compared with those who do not receive chemoimmunotherapy, patients with resectable NSCLC who receive chemoimmunotherapy before surgery may achieve higher major pathological response (MPR) and pathological complete response (pCR) rates, and significantly improved survival (2, 3, 5). In the phase III CheckMate 816 study, compared with neoadjuvant chemotherapy alone, respectively, chemoimmunotherapy improved the pCR rate from 2% to 24%, and the MPR also improved (37% vs 9%). Furthermore, median events-free survival was extended from 20.8 to 31.6 months with chemoimmunotherapy (5). Several large-scale real-world analyses also confirmed the positive role of neoadjuvant chemoimmunotherapy in the treatment of resectable NSCLC (6, 7). As a result, neoadjuvant chemoimmunotherapy has been established as the optimal choice in several guidelines and expert consensuses (2, 8–10).

However, controversies remain regarding the use of neoadjuvant chemoimmunotherapy for NSCLC, which is increasing rapidly in clinical practice. The optimal number of neoadjuvant therapeutic cycles is frequently debated and has not been fully elucidated. In a systematic review that evaluated 10 prospective clinical trials of neoadjuvant chemoimmunotherapy, two to four cycles of neoadjuvant therapy were administered, and surgery was performed safely with excellent efficacy and an acceptable incidence of adverse events (AEs) (4). In a prospective phase II clinical trial (neoSCORE), three cycles of neoadjuvant sintilimab plus chemotherapy achieved a 14.5% increase in the MPR rate compared with two cycles, but the difference was not statistically significant (11). In a recently published international expert consensus, three cycles of neoadjuvant chemoimmunotherapy were recommended on the basis of data from the CheckMate 816 study (2). Deng et al. reported that three and four cycles of neoadjuvant chemoimmunotherapy in stage III NSCLC might achieve higher MPR rates compared with two cycles in patients classified as clinical complete/partial response (CR/PR) (p=0.081) (12). In other real-world analyses, one to five cycles of neoadjuvant chemoimmunotherapy were also reported; however, the clinical differences between the therapeutic cycles were not mentioned in these studies (6, 13–15). Therefore, the neoadjuvant therapeutic efficacy after different numbers of cycles requires further investigation. Furthermore, the impact on surgery and possible perioperative difficulties following different numbers of therapeutic cycles should also be identified.

In the present study, the perioperative outcomes of pulmonary resection following neoadjuvant PD-1 inhibitors plus chemotherapy in resectable NSCLC were retrospectively analyzed. The primary aim was to determine the optimal number of neoadjuvant therapeutic cycles. Related AEs and postoperative morbidities were also compared, and potential predictive biomarkers for therapeutic efficacy were explored.

## Materials and methods

### Patients

This study was a real-world clinical analysis that retrospectively identified all patients who received neoadjuvant chemoimmunotherapy and underwent surgery at Hunan Cancer Hospital from January 2020 to August 2022. This study was performed in accordance with the Declaration of Helsinki (as revised in 2013) and was approved by the Ethics Committee of Hunan Cancer Hospital (2023032), with written informed consent obtained from the patients.

The inclusion criteria were (1) age 18–80 years; (2) primary treated NSCLC (clinical stage IB–IIIB: stage IB–IIB, which comprised T2–3N0 and T1–2N1; stage IIIA, which comprised T1–2N2, T3N1, and T4N0–1; and stage IIIB, which comprised T3–4N2M0); (3) resectable lung cancer; (4) Karnofsky physical status score ≥ 80; and (5) received at least two cycles of neoadjuvant chemoimmunotherapy. Patients were excluded if they met the following criteria: (1) participated in a double-blind clinical trial; (2) EGFR/ALK mutation-positive; (3) N3 or distant metastases; (4) received more than four cycles of neoadjuvant therapy; (5) patient refused or could not tolerate surgery; and (6) incomplete medical records.

### Treatment modality

All patients received two to four cycles of neoadjuvant chemoimmunotherapy. The patients were divided into two groups on the basis of the number of therapeutic cycles as a 2-cycle group and 3-4-cycles group. The chemotherapy regimens were: platinum-based drugs combined with paclitaxel or vinorelbine for patients with squamous cell carcinoma and platinum-based drugs combined with pemetrexed for patients with non-squamous cell carcinoma (21 days per cycle), with all regimens in accordance with the National Comprehensive Cancer Network guidelines and Chinese lung cancer treatment guidelines (16, 17). PD-1 inhibitors used intravenously in accordance with international consensus comprised sintilimab, toripalimab, camrelizumab, pembrolizumab, nivolumab, and tislelizumab.

After a multidisciplinary discussion, curative-intent resection was performed after recovery from neoadjuvant treatment. Surgery performed beyond 42 days after the final cycle of neoadjuvant therapy was defined as delayed surgical resection. Surgical approaches comprised video-assisted thoracic surgery, and conversion and open thoracotomy. The scope of resection comprised lobar, bilobar, and whole-lung. Additionally, some cases underwent sleeve resection.

### Clinical and pathological features

The clinical data included in this study were as follows: age, sex, smoking, weight loss at initial diagnosis (weight loss of more than 5% in the six months prior to the initial visit), pathological type, tumor length before therapy, tumor location, clinical stage, pathological stage, pathological response, AEs associated with neoadjuvant therapy, and postoperative complications. All patients underwent computed tomography (CT) at the initial diagnosis, after the second cycle of neoadjuvant therapy, and before surgery (only for patients who received at least three cycles of neoadjuvant therapy) to assess the tumor changes. Clinical response assessment was in accordance with the Response Evaluation Criteria in Solid Tumors (RECIST v1.1), as follows: partial remission (PR) was defined as a reduction of > 30% in the longest diameter of the tumor from its initial size. Progressive disease was defined as an increase in the longest diameter of the tumor of > 20% from the minimum recorded value, an absolute increase of at least 5 mm, the appearance of new lesions, or definite progression of non-target lesions. Stable disease was defined as meeting neither PR nor progressive disease criteria (18).

PD-L1 expression in the primary tumor at initial diagnosis was evaluated using the Dako murine 22C3 anti-human PD-L1 antibody (Agilent Corp., Denmark) and determined by the tumor proportion score (TPS). Postoperative paraffin-embedded pathological tissues were evaluated by two trained pathologists to clarify tumor remission. Pathological CR (pCR) was defined as the absence of live tumor cells in the surgically-resected specimen, and the MPR rate was defined as < 10% viable tumor cells remaining (19).

### Statistical analysis

The primary endpoint was the MPR rate, and the secondary endpoints were the rate of delayed surgery, radical resection (R0), AEs associated with neoadjuvant therapy, perioperative morbidity, and 1-year disease-free survival (DFS). DFS was defined as the time (in months) from the date of surgery to the date of confirmed disease progression or death. The correlation between postoperative pathological remission and the degree of tumor regression was assessed by imaging. PD-L1 expression was also evaluated.

All statistical analyses were performed using R 4.2.1 (www.r-project.org). Continuous data were expressed as mean ± standard deviation and analyzed by the two-tailed t-test or rank sum test. Categorical data were expressed as frequencies and percentages (%) and analyzed by the chi-square test or Fisher’s exact test. Cox proportional risk regression models were used to determine the factors affecting DFS. Survival analysis was performed using the Kaplan–Meier method, and differences between survival curves were compared by the log-rank test. Stratified optimal thresholds were obtained by plotting the receiver operating characteristic curves and calculating the Youden index (20). Statistical significance was set at p < 0.05 (two-sided).

## Results

### Baseline clinical characteristics of the patients

**Figure 1:**
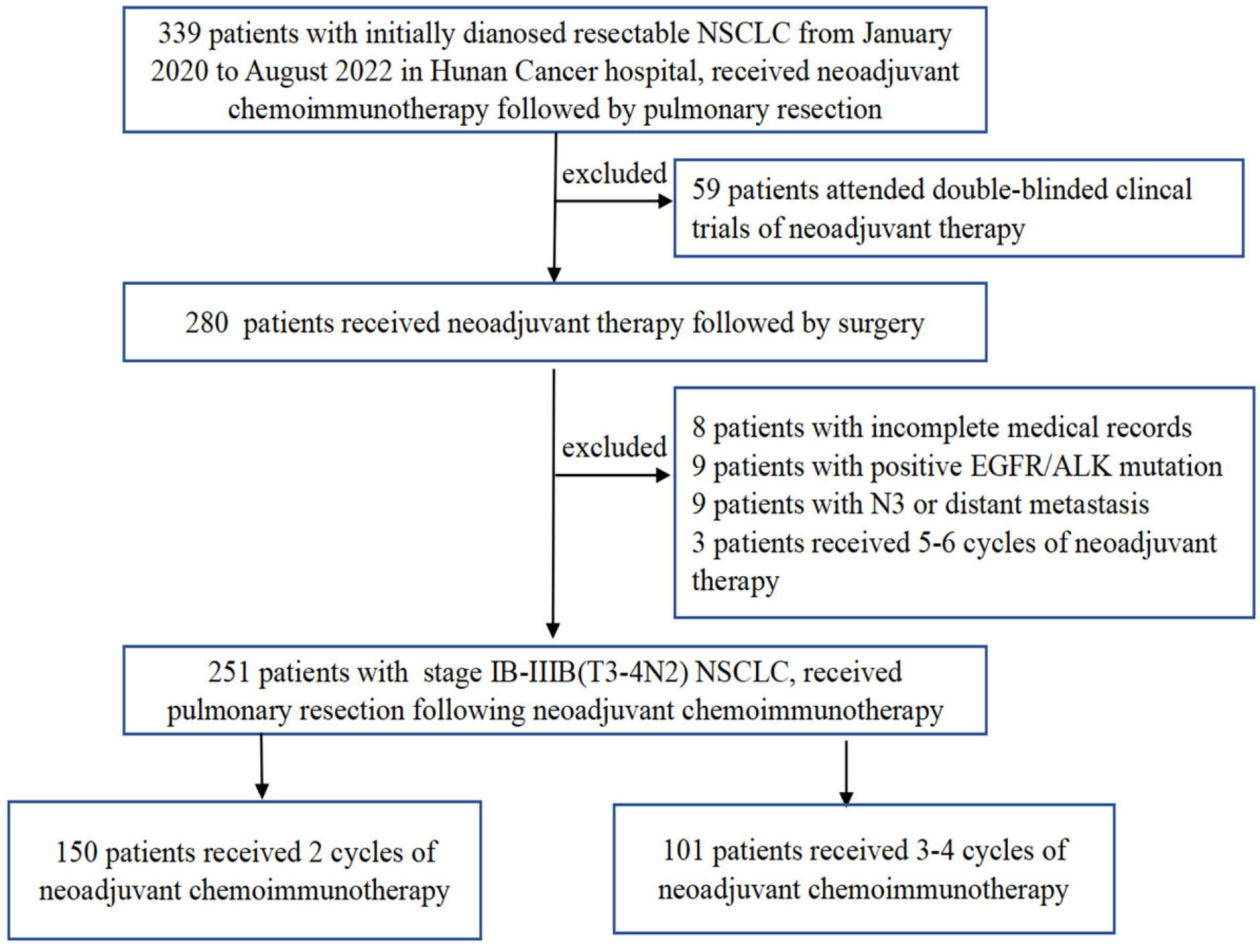
Flow chart of patient selection.

A total of 251 patients were included in the present study (**Figure 1**). The preoperative clinical stage was stage IB–IIB in 56 (22.3%), stage IIIA in 106 (42.2%), and stage IIIB in 89 (35.5%). There were 237 males and 14 females in the entire cohort, with a median age of 60 years (range, 41–78 years). Weight loss occurred after presentation in 39 cases (15.6%), and there was a history of smoking in 233 cases (92.8%). The average length of the primary tumor at diagnosis was 5.05 ± 1.79 cm (range, 1.4 cm–12.0 cm), with 216 cases of central lung cancer (86.0%) and 35 cases of peripheral lung cancer (14.0%). Regarding the pathology, squamous cell cancer was the predominant pathological type (n = 207) and accounted for 82.5% of all cases.

On the basis of the number of neoadjuvant treatment cycles before surgery, patients were divided into a 2-cycle group, which comprised 150 cases (59.8%), and a 3-4-cycles group, which comprised 68 cases (27.1%) who received three cycles and 33 cases who received four cycles (13.1%) of treatment. As summarized in **Table 1**, none of the baseline characteristics were statistically different between the two groups.

**Table 1:** Baseline characteristics for 251 NSCLC patients received neoadjuvant PD-1 inhibitors plus chemotherapy.

Characteristics		Number of Neoadjuvant therapy cycles (%)		P value
		2 (n=150)	3-4 (n=101)	
Age	`x ± s ^a,^ y	59.87 ± 6.99	59.68 ± 7.31	0.841
Gender	Male	144 (96.0)	93 (92.1)	0.184
	Female	6 (4.0)	8 (7.9)	
Smoking habits	Non-smoker	11 (7.3)	9 (8.9)	0.651
	Present/ex-smoker	139 (92.7)	92 (91.1)	
Weight loss at initial diagnosis	Yes	23 (15.3)	16 (15.8)	0.913
	No	127 (84.7)	85 (84.2)	
Pathological type	SCC	124 (82.7)	83 (82.2)	0.920
	Non-SCC	26 (17.3)	18 (17.8)	
Tumor length before therapy	`x ± s ^a,^ cm	5.12 ± 1.67	4.94 ± 1.96	0.441
Tumor location	Peripheral	23 (15.3)	12 (11.9)	0.439
	Central	127 (84.7)	89 (88.1)	
cT stage	T1-3	117 (78.0)	82 (81.2)	0.541
	T4	33 (22.0)	19 (18.8)	
cN stage	N0	10 (6.7)	5 (5.0)	0.842
	N1	54 (36.0)	36 (35.6)	
	N2	86 (57.3)	60 (59.4)	
cTNM stage	IB- IIB	35 (23.3)	21 (20.8)	0.885
	IIIA	63 (42.0)	43 (42.6)	
	IIIB	52 (34.7)	37 (36.6)	
PD-1 inhibitors	Sintilizumab	45 (30.0)	29 (28.7)	0.238
	Toripalimab	29 (19.3)	21 (20.8)	
	Camrelizumab	23 (15.3)	9 (8.9)	
	Pembrolizumab	22 (14.7)	18 (17.8)	
	Nivolumab	15 (10.0)	18 (17.8)	
	Tislelizumab	16 (10.7)	6 (5.9)	

a Variables were described by mean (x) and standard deviation(s).

NSCLC, non-small cell lung cancer; SCC, squamous cell cancer; cT, clinical T stage before treatment; cN, clinical N stage before treatment; cTNM, clinical TNM stage before treatment, stage IB-IIB include T2-3N0, T1-2N1, stage IIIA include T1-2N2, T3N1, T4N0-1, and stage IIIB include T3-4N2M0.

### Surgical outcome after neoadjuvant chemoimmunotherapy

Of the total number of patients, 109 underwent video-assisted thoracic surgery (43.4%), 81 underwent conversion operation (32.3%), and 61 underwent thoracotomy (24.3%). Regarding the extent of resection, 196 underwent lobectomy (78.1%), 38 underwent bilobectomy (15.1%), 15 underwent pneumonectomy (6.0%), and two underwent exploratory procedures (0.8%). Radical resection (R0) was achieved in 234 of the total number of patients (93.2%). Regarding complex surgery, 101 patients underwent sleeve resection (40.2%), including 3 vascular sleeve resections (1.2%), 76 bronchial sleeve resections (30.3%), and 22 vascular + bronchial sleeve resections (8.8%). Nineteen patients required postoperative treatment in the intensive care unit (7.6%). The average postoperative hospital stay was 6.3 days. The postoperative pathological stages were stage 0 (96 cases, 38.2%), stage I (66 cases, 26.3%), stage II (42 cases, 16.7%), and stage III (47 cases, 18.7%). Regarding pathological remission, there were 144 MPRs (57.4%), including 96 pCRs (38.2%); 205 patients received post-operative adjuvant therapy (81.7%).

As shown in **Table 2**, 93.3% of all cases in the 2-cycle group achieved radical (R0) resection, with approximately 93.1% in the 3-4-cycles group (p=0.961). The MPR rate in the 2-cycle group was 57.3% and not significantly different from that of 57.4% in the 3-4-cycles group (p=0.529). All other factors did not differ between the two treatment groups, except for the interval between neoadjuvant treatment and surgery. Thirty-two patients (31.7%) in the 3-4 cycles group received surgery > 42 days after the final cycle of neoadjuvant therapy, significantly more than the 24 patients (16.0%) in the 2-cycle group (p=0.003).

**Table 2:** Correlation between neoadjuvant therapeutic cycles and surgical outcomes.

Characteristics		Number of Neoadjuvant therapy cycles (%)		P value
		2 (n=150)	3-4 (n=101)	
Interval time between final neoadjuvant therapy and surgery	≤ 42day	126 (84.0)	69 (68.3)	0.003
	> 42day	24 (16.0)	32 (31.7)	
Surgical approach	VATS	67 (44.7)	42 (41.6)	0.288
	Conversion	43 (28.7)	38 (37.6)	
	Thoracotomy	40 (26.7)	21 (20.8)	
Surgical radicality	Radical	140 (93.3)	94 (93.1)	0.961
	Palliative	9 (6.0)	6 (5.9)	
	Exploration	1 (0.7)	1 (1.0)	
Extent of resection	Lobectomy	117 (78.0)	79 (78.2)	0.934
	Bilobectomy	22 (14.7)	16 (15.8)	
	Pneumonectomy	10 (6.7)	5 (5.0)	
	Unremoved	1 (0.7)	1 (1.0)	
Sleeve resection	No	95 (63.3)	55 (54.5)	0.384
	Bronchial/bronchoplasty	43 (28.7)	33 (32.7)	
	Vascular/angioplasty	2 (1.3)	1 (1.0)	
Bronchial+Vascular	10 (6.7)	12 (11.9)	
Postoperative ICU admission	Yes	13 (8.7)	6 (5.9)	0.423
	No	137 (91.3)	95 (94.1)	
Hospital stays after surgery	Median (range), days	6.5 (3, 30)	6.0 (4, 21)	0.122
Pathological response	MPR	86 (57.3)	58 (57.4)	0.529
	PR	41 (27.3)	32 (31.7)	
	SD+PD	23 (15.3)	11 (10.9)	
ypT stage	T0-2	132 (88.0)	96 (95.0)	0.058
	T3-4	18 (12.0)	5 (5.0)	
ypN stage	N0	109 (72.7)	67 (66.3)	0.424
	N1	24 (16.0)	17 (16.8)	
	N2	17 (11.3)	17 (16.8)	
ypTNM stage	0	57 (38.0)	39 (38.6)	0.967
	I	41 (27.3)	25 (24.8)	
	II	25 (16.7)	17 (16.8)	
	IIIA-IIIB	27 (18.0)	20 (19.8)	
Adjuvant therapy	Yes	126 (84.0)	79 (78.2)	0.246
	No	24 (16.0)	22 (21.8)	

ypT, pathological T stage after treatment; ypN, pathological N stage after treatment; ypTNM, pathological TNM stage after treatment; VATS, video-assisted thoracic surgery; MPR, major pathological response; PR, partial remission; SD, stable disease; PD, progressive disease.

### AEs and postoperative complications

A total of 160 patients experienced AEs following neoadjuvant chemoimmunotherapy (63.7%), with hepatic dysfunction and myelosuppression as the most common categories at 59 (23.5%) and 47 (18.7%) cases, respectively. Regarding the Common Terminology Criteria for Adverse Events grade, 89 (35.5%) cases were grade I, 39 (15.5%) were grade II, 23 (9.2%) were grade III, 19 (7.6%) were grade IV, and there were no grade V AEs. Additionally, 52 (20.7%) patients experienced weight loss during neoadjuvant therapy. The incidence of AEs was higher with 3–4 cycles of neoadjuvant therapy compared with two cycles of neoadjuvant therapy (72.3% versus 58.0%, respectively; p=0.021); however, there was no significant difference in the type (p=0.434) and severity of AEs (p=0.869) (**Table 3**).

**Table 3:** The adverse events of neoadjuvant therapy.

Characteristics	Number of Neoadjuvant therapy cycles (%)		*P* value
	2 (n=150)	3-4 (n=101)	
**Adverse events**			
No	63 (42.0)	28 (27.7)	0.021
Yes	87 (58.0)	73 (72.3)	
**CTCAE grade**			
Any grade	N=87	N=73	0.869
I	48 (55.2)	41 (56.2)	
II	23 (26.4)	16 (21.9)	
III	9 (10.3)	10 (13.7)	
IV	7 (8.0)	6 (8.2)	
V	0	0	
**Adverse event types**	N=87	N=73	0.434
Hepatic dysfunction	35 (40.2)	24 (32.9)	
Myelosuppression	25 (28.7)	22 (30.1)	
Thyroid dysfunction	11 (12.6)	12 (16.4)	
Renal dysfunction	6 (6.9)	10 (13.7)	
Gastrointestinal reaction	6 (6.9)	1 (1.4)	
Pneumonia	1 (1.1)	0	
Myocarditis	1 (1.1)	0	
Erythra	1 (1.1)	2 (2.7)	
Numbness of extremities	1 (1.1)	1 (1.4)	
Myositis	0	1 (1.4)	

CTCAE, Common Terminology Criteria for Adverse Events.

Postoperative complications occurred in 55 patients, accounting for 21.9% of all patients. The most common complications were pneumonia and prolonged air leak, which occurred in 26 (10.4%) and 10 (4.0%) cases, respectively. Two patients died within 30 days postoperatively. **Table 4** shows the postoperative complications associated with two and three or more cycles of neoadjuvant therapy. Two cycles of therapy had a higher rate of postoperative complications vs three or more cycles (28.0% versus 12.9%, respectively; p=0.004); however, there was no significant difference in the type of complications (p=0.192). Four patients developed arrhythmia/cardiac failure, two developed immunologic hepatitis, and one developed immunologic myocarditis after surgery in the 2-cycle group, while no such complications were observed in the 3-4-cycles group.

**Table 4:** Postoperative complications after surgery and mortality within 30 days.

Characteristics	Number of Neoadjuvant therapy cycles (%)		*P* value
	2 (n=150)	3-4 (n=101)	
Postoperative complications			
Yes	42 (28.0)	13 (12.9)	0.004
No	108 (72.0)	88 (87.1)	
**Complication types**	n=42	n=13	0.192
Pneumonia	21 (50.0)	5 (38.5)	0.467
Prolonged air leak	8 (19.0)	2 (15.4)	1
Arrhythmia/Cardiac failure	4 (9.5)	0	0.562
Chyle	1 (2.4)	2 (15.4)	0.136
Gastrointestinal complications	2 (4.8)	1 (7.7)	0.562
Urinary retention	0	2 (15.4)	0.053
Respiratory failure	1 (2.4)	0	1
Hoarseness	1 (2.4)	0	1
Hepatic dysfunction	1 (2.4)	1 (7.7)	0.420
Immunologic hepatitis	2 (4.8)	0	1
Immunologic myocarditis	1 (2.4)	0	1
30-day mortality			
No	148 (98.7)	101 (100.0)	0.244
Yes	2 (1.3)	0	

### Survival analysis

**Figure 2:**
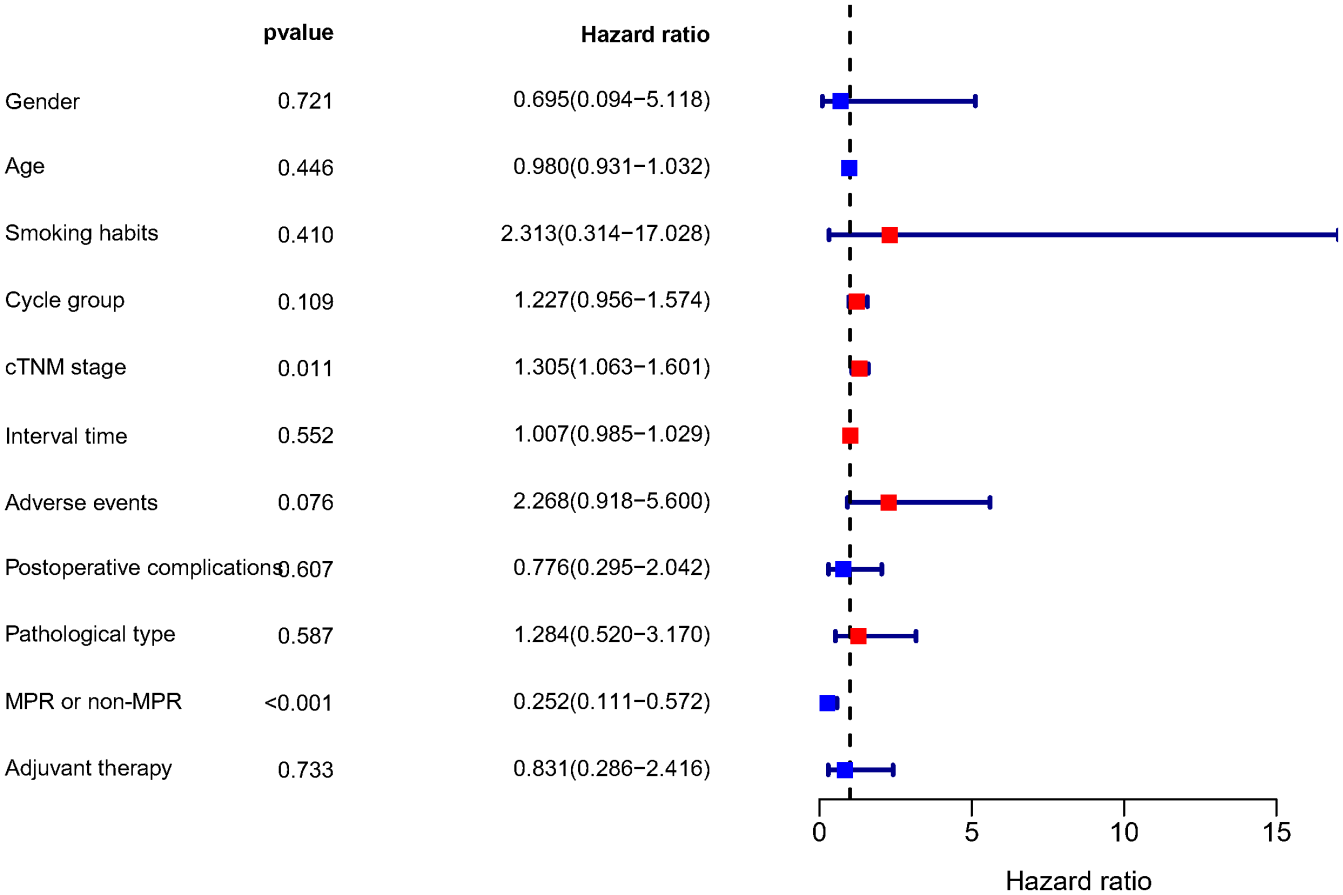
Results of the univariate COX regression analysis of DFS. DFS, disease-free survival; Cycle group, Neoadjuvant treatment cycle grouping; cTNM, clinical TNM stage; MPR, major pathological response.

**Figure 3:**
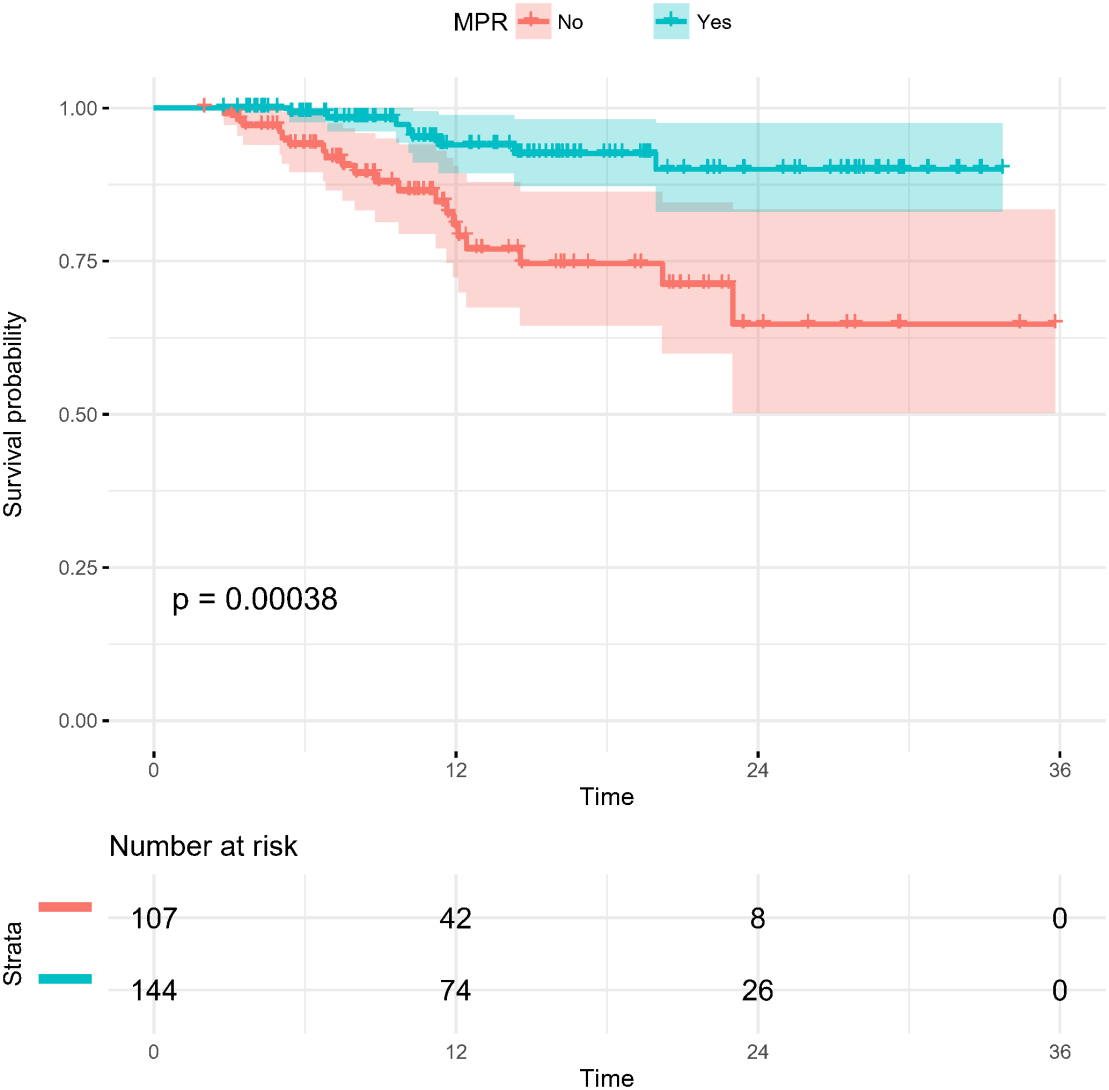
Kaplan-Meier survival curves of disease-free survival in patients stratified by pathological response (MPR and non-MPR). MPR, major pathological response.

To 30 November 2022, the median follow-up time in this study was 12.55 months, and we chose to include DFS as an observational endpoint. As shown in **Figure 2**, univariate COX regression analysis showed that of the factors included in the analysis, only preoperative clinical tumor-node-metastasis stage (hazard ratio (HR): 1.305, 95% confidence interval (CI): 1.063–1.601; p=0.011) and achieving MPR (HR: 0.252, 95% CI: 0.111–0.572; p<0.001) affected DFS. However, no correlation between the number of neoadjuvant therapeutic cycles and DFS was identified (p=0.109). Kaplan–Meier curves showed significantly higher DFS for patients who achieved MPR, with a 1-year DFS of 94.0%, significantly higher than that of 80.9% in non-MPR patients (p<0.001) (**Figure 3**).

### Tumor regression on imaging correlated with pathological remission

**Figure 4:**
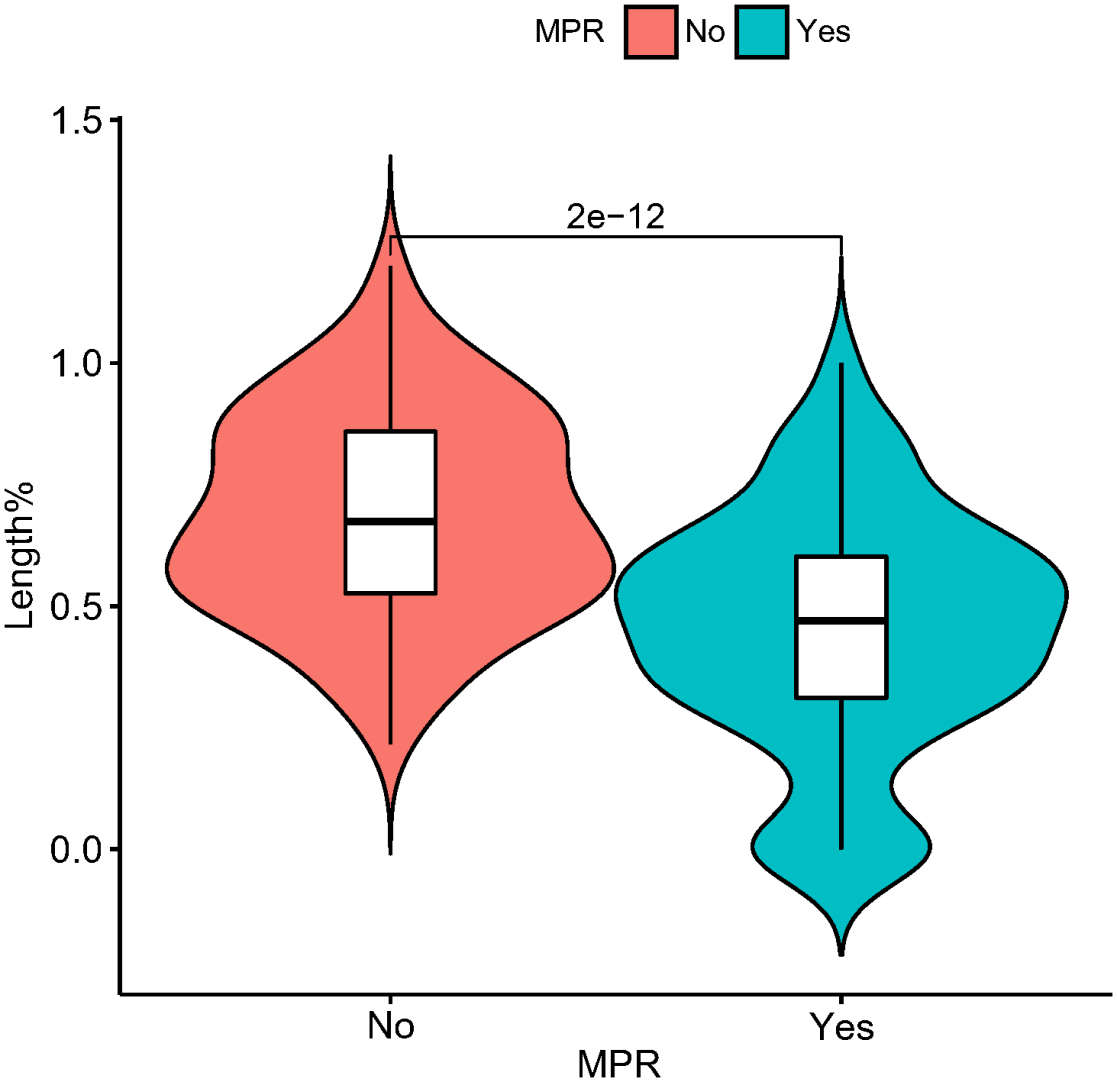
Violin plot demonstrating the relationship between pathological remission (MPR or non-MPR) and tumor length (as a percentage at diagnosis) after second neoadjuvant treatment with CT imaging. In this figure, the horizontal coordinate is the presence or absence of MPR, and the vertical coordinate is the percentage of tumor length diameter after the second neoadjuvant treatment at the first diagnosis. MPR, major pathological response.

**Figure 5:**
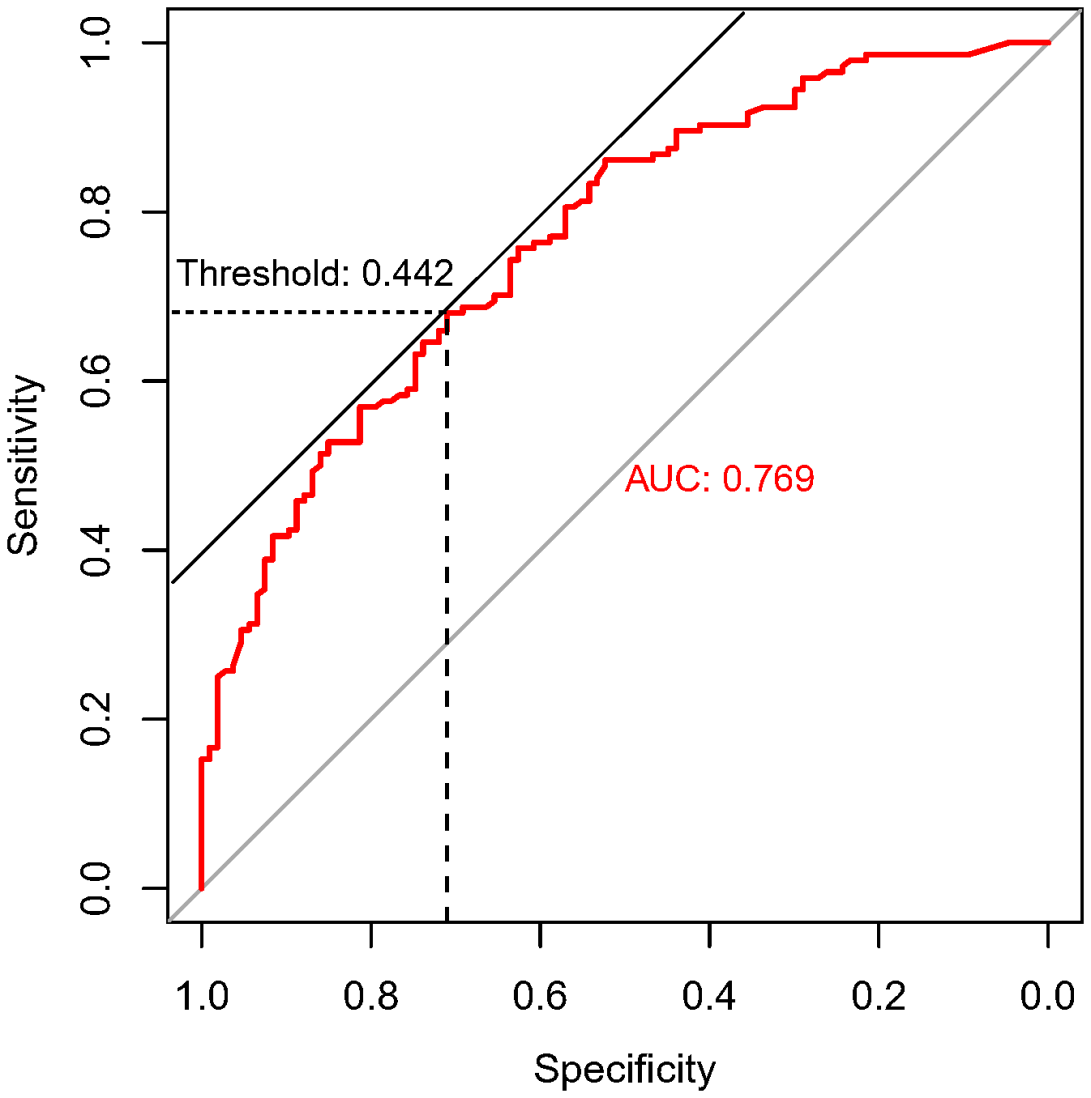
ROC curve of maximum diameter shortening% of tumor predicting pathological remission. This graph demonstrates the specificity and sensitivity of the degree of tumor imaging regression in predicting MPR at different cut-off values. The horizontal coordinate is the specificity, and the vertical coordinate is the sensitivity. The area under the ROC curve (AUC) is 0.769, and the tangent position between the 45-degree diagonal line and the ROC curve is the maximum Youden index, and the threshold value is 0.442, which means the tumor length diameter is 44.2% smaller than that at the time of diagnosis after the second cycle of neoadjuvant treatment.

**Figure 6:**
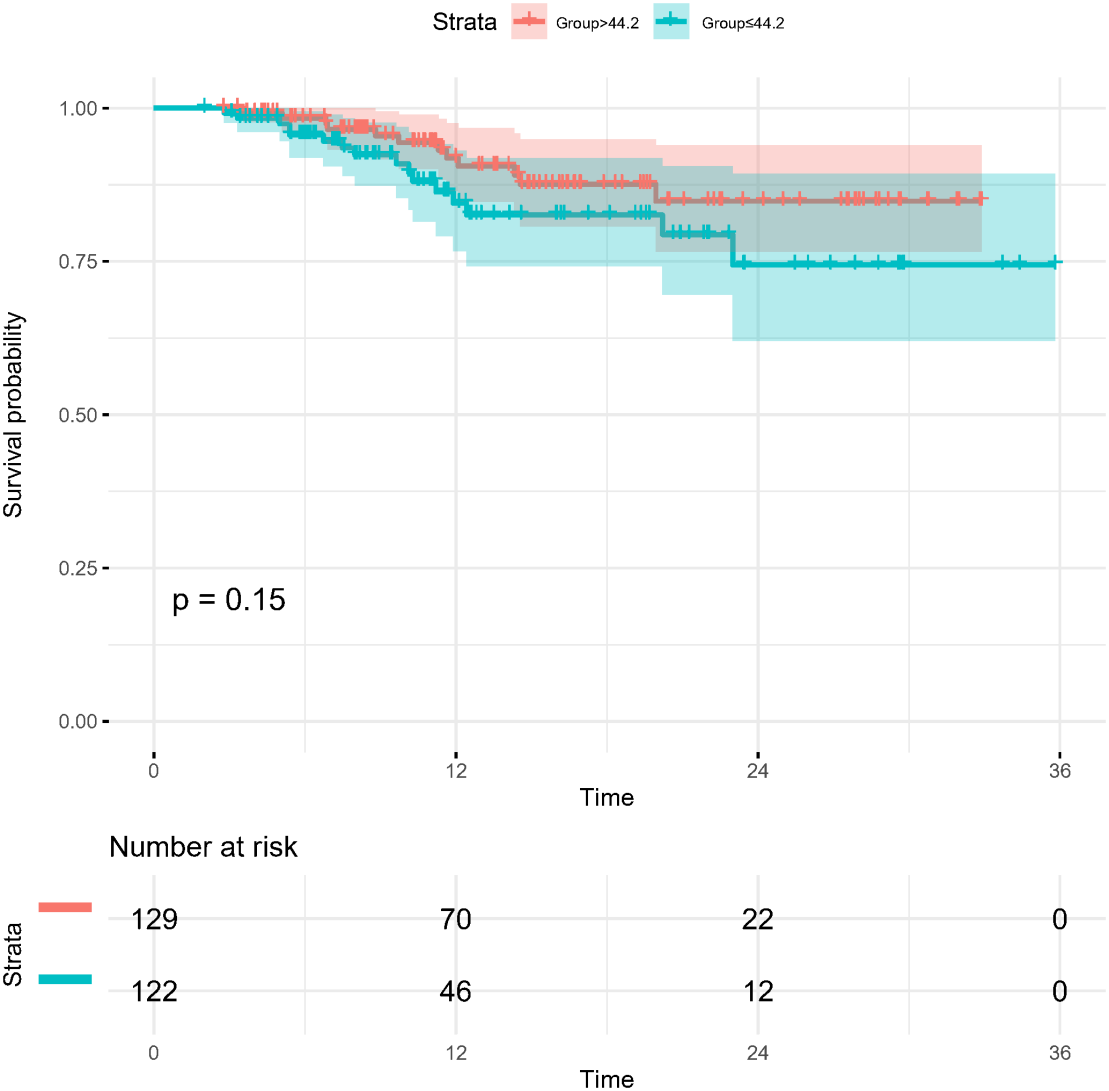
Kaplan-Meier survival curves for disease-free survival for patients grouped by imaging assessment of the degree of tumor regression (> 44.2% and ≤44.2% reduction in long diameter).

In the present study, each patient underwent tumor re-evaluation by CT after the second cycle of neoadjuvant therapy. Sixteen patients achieved radiographic CR (6.4%), 169 achieved PR (67.3%), 65 achieved stable disease (25.9%), and 1 achieved progressive disease (0.4%). After the completion of all cycles of neoadjuvant therapy, radiographic re-evaluation by CT detected 21 CR cases (8.4%), 177 PR cases (70.5%), and 53 stable disease cases (21.1%). In a further analysis, we found a correlation between the degree of tumor regression assessed by CT after the second cycle of neoadjuvant treatment and postoperative pathological remission, for patients with MPR with more radiographic tumor regression in diameter after two-cycle neoadjuvant treatment (p<0.001) (**Figure 4**). Plotting the receiver operating characteristic curve and calculating the Youden index, we divided the cases into two groups using 44.2% tumor maximum diameter regression as the threshold (**Figure 5**). The area under the curve was 0.769. A total of 129 patients (51.4%) had > 44.2% reduction in tumor diameter on CT assessment after 2-cycle neoadjuvant treatment. The chi-square test revealed that for the group with regression rates ≤ 44.2%, the number of neoadjuvant treatment cycles was associated with pathological remission rates, with more treatment cycles associated with higher MPR rates (29.9% versus 47.3%; 2 cycles vs 3-4 cycles, respectively; p=0.048) (**Table 5**). In contrast, for the group with regression rates > 44%, more treatment cycles (3, 4) were not associated with the MPR rate (69.6% versus 79.5%; 2 cycles vs 3-4 cycles, respectively; p=0.205, **Table 5**). Additionally, the Kaplan–Meier curves showed that the 1-year DFS appeared to be better in cases with > 44.2% diameter reduction; however, the result was not statistically significant (94.6% versus 89.3%; > 44.2% vs ≤ 44.2%, respectively; P=0.150) (**Figure 6**).

**Table 5:** Relationship between the degree of tumour shrinkage assessed by imaging and the rate of pathological response after different cycles of treatment.

Characteristics	Number of Neoadjuvant therapy cycles (%)		*P* value
	2 (n=150)	3-4 (n=101)	
**> 44.2% reduction in tumor length**	n=83	n=46	0.205
MPR	66 (79.5)	32 (69.6)	
Non-MPR	17 (20.5)	14 (30.4)	
**≤44.2% reduction in tumor length**	n=67	n=55	0.048
MPR	20 (29.9)	26 (47.3)	
Non-MPR	47 (70.1)	29 (52.7)	

MPR, major pathological response.

### PD-L1 expression correlated with pathological remission

The correlation between PD-L1 expression at initial diagnosis in the primary tumor and pathological response was further explored in the present study. There were 66 cases with complete PD-L1 expression information. As shown in **Table 6**, for cases with TPS ≤ 10%, more cycles of neoadjuvant treatment increased the MPR rate (37.5% versus 9.5%; 3-4 cycles vs 2 cycles, respectively; p=0.041). However, for cases with TPS > 10%, no statistically significant difference was observed between 2 cycles and 3-4 cycles of neoadjuvant treatment (p=0.518).

**Table 6:** Relationship between the PD-L1 expression and the rate of pathological remission after different cycles of treatment.

PD-L1 expression (TPS)	Number of Neoadjuvant therapy cycles (%)		*P* value
	2 (n=35)	3-4 (n=31)	
≤ 10**%**	n=21	n=16	0.041
MPR	2 (9.5)	6 (37.5)	
Non-MPR	19 (90.5)	10 (62.5)	
> **10%**	n=14	n=15	0.518
MPR	10 (71.4)	9 (60.0)	
Non-MPR	4 (28.6)	6 (40.0)	

MPR, major pathological response; TPS, tumor proportion score (PD-L1 expression).

## Discussion

In the present study, we retrospectively analyzed the clinical data for patients who received 2–4 cycles of neoadjuvant chemoimmunotherapy, with several interesting findings, as follows: First, compared with 2-cycle neoadjuvant therapy, the MPR rate did not increase significantly after 3-4 cycles of therapy, although more patients in the 3-4-cycles group underwent surgery > 42 days after the final cycle of neoadjuvant therapy. Moreover, no significant difference was identified in the R0 resection rate and 1-year DFS between the two treatment groups. Second, AEs related to neoadjuvant therapy were observed more frequently in the 3-4-cycles group compared with the 2-cycle group, while the morbidity of postoperative complications was lower after 3-4 cycles of therapy. Third, more cycles of neoadjuvant therapy improved the MPR rate in patients with ≤ 44.2% tumor regression assessed by CT after the second cycle of neoadjuvant therapy or TPS ≤ 10% in the primary tumor at the initial diagnosis.

The choice of the number of cycles of neoadjuvant chemoimmunotherapy for NSCLC is very important to balance the benefits of neoadjuvant therapy with the associated AEs (21). In a mice model study, more cycles of immunotherapy did not increase the efficacy; however, immune-related AEs (irAEs) did increase (21). The authors suggested that the underlying mechanism is that insufficient treatment time does not fully activate tumor-specific T cells, while excessive treatment leads to dysregulation/depletion of these cells. Furthermore, optimal efficacy can be achieved only by timely removal of the primary tumor at the peak of tumor-specific T cell expansion (21). As there is no convincing comparison in the perioperative outcomes and pathological responses after different numbers of cycles of neoadjuvant chemoimmunotherapy for NSCLC, the optimal number is unknown. Three cycles of neoadjuvant chemoimmunotherapy were given in the CheckMate 816 and SAKK 16/14 studies; however, 7%–9.7% of the patients failed to undergo pulmonary resection owing to tumor progression and 21% underwent delayed surgery (5, 22), indicating that 3-cycle neoadjuvant therapy was not optimal in certain patients. In these prospective studies, 2–4 cycles of neoadjuvant therapy were planned before treatment was initiated, and patients’ responses and AEs were not considered in the selection of the number of therapeutic cycles. Therefore, the problem remains whether the number of neoadjuvant therapeutic cycles could be changed on the basis of patients’ responses to achieve a better balance between the advantages and disadvantages related to the treatment.

In the present study with a large sample size, MPR was considered the primary endpoint as in other clinical trials of neoadjuvant immunotherapy (2, 23). There were 144 MPR cases (57.4%), including 96 pCR cases (38.2%) identified in our analysis, similar to MPR rates ranging from 20% to 74% in previous reports (4, 14). In our study, MPR was also correlated with better DFS. Unfortunately, no increase in the MPR rate was observed after more cycles of neoadjuvant chemoimmunotherapy in our analysis. A phase II prospective clinical trial demonstrated that a higher MPR rate might be achieved after three cycles of neoadjuvant chemoimmunotherapy compared with after two cycles; however, statistical significance was not reached owing to the limited sample size (11). In a recent retrospective study by Deng et al., three or four cycles of neoadjuvant treatment did not significantly increase MPR rates compared with two cycles for the entire group, while MPR rates for two, three, four, five or more cycles were 43.8%, 71.0%, 71.4%, and 33.3%, respectively, in patients classified as clinical CR/PR (p=0.081). Notably, there may have been bias in the study in that patients who were more sensitive to neoadjuvant chemoimmunotherapy tended to undergo radical pulmonary resection earlier compared with non-sensitive patients, and neoadjuvant treatment might have been stopped during the therapeutic period after fewer cycles. Therefore, prospective clinical trials with larger sample sizes are required to verify whether more cycles of neoadjuvant chemoimmunotherapy could improve the pathological response rate.

AEs and postoperative morbidity are major concerns during the administration of neoadjuvant chemoimmunotherapy. In a systematic analysis by Jiang et al, the pooled incidence of treatment-related AEs and severe AEs after neoadjuvant chemoimmunotherapy was 73.9% and 18.0%, respectively (4), similar to the rates of 63.7% and 16.8% (Common Terminology Criteria for Adverse Events grade III–IV) in the present study. Previous randomized clinical trials have demonstrated that neoadjuvant chemoimmunotherapy has no prominent impact on surgical operation and its safety compared with chemotherapy alone (2, 4, 5). However, our analysis detected that more neoadjuvant therapy-related AEs were observed in the 3-4-cycles group compared with the 2-cycle group, while the incidence of postoperative complications was lower in the former group.3-4 Besides, as reported by Martins et al, most main irAEs manifest within 4–15 weeks after initiation of PD1/PD-L1 therapy (24), and combined therapy might have an earlier onset of AEs. In the 2-cycle group in the present study, surgery was performed within 7–9 weeks after the first cycle of therapy, in accordance with the schedule, compared with 10–15 weeks or later in the 3-4-cycles group. Therefore, we suspect that increasing the number of neoadjuvant therapy cycles to 3-4 may improve the safety of pulmonary resection via reducing potential perioperative irAEs.

According to the Response Evaluation Criteria in Solid Tumors (RECIST 1.1), CT is a routine radiographic modality to assess therapeutic response in NSCLC (18). However, in 41%–45% of patients, the pathological response was inconsistent with the CT findings after neoadjuvant chemoimmunotherapy (2, 25, 26). Regarding the relationship between imaging assessment of tumor remission and MPR, the preoperative radiographic CR rate in our study was only 8.4%, while the MPR rate of pathological assessment was 57.6%. In the CheckMate 159 trial, the CR rate of imaging assessment was 10%, while the MPR rate of pathological assessment was approximately 45% (27). There is a large discrepancy between pathological response and imaging remission. Although a previous study concluded that response assessed by CT (RECIST) was unrelated to MPR (28), the deep learning score based on CT findings within 2 weeks preceding neoadjuvant administration effectively predicted MPR in NSCLC patients, with an area under the curve of 0.72 (14). In our study, more cycles of neoadjuvant therapy improved the MPR rate in patients with ≤ 44.2% tumor regression in diameter assessed by CT after the second cycle of neoadjuvant therapy. As a result, the degree of tumor shrinkage assessed on CT during neoadjuvant chemoimmunotherapy may be a valuable indicator to guide the selection of the number of treatment cycles.

Several prospective clinical trials reported that patients with positive PD-L1 expression (TPS ≥ 1%) had significantly better MPR and pCR rates compared with those with negative PD-L1 expression (9). However, the relationship between the number of therapeutic cycles and PD-L1 expression was analyzed rarely in previous studies. In the present study, TPS was correlated with the efficacy of the number of therapeutic cycles, and more cycles of neoadjuvant therapy improved the MPR rate in patients with TPS ≤ 10% in the primary tumor at the initial diagnosis. However, TPS values were available for only 66 patients; therefore, the sample size was too small to draw a firm conclusion, and further validation is required.

This study has the following limitations. First, owing to the retrospective design, although the baseline characteristics were not significantly different between the two groups, selection bias is possible, and a randomized controlled clinical trial is needed to verify the results. Second, data for PD-L1 expression were recorded in only a small number of patients. Third, owing to the limited sample size, patients receiving three and four cycles of neoadjuvant therapy were incorporated into one group, and differences between those receiving three vs four cycles of treatment remain unknown. Finally, the follow-up period of this study was short, and the survival difference requires further investigation.

In conclusion, our data support the positive role of chemoimmunotherapy in the neoadjuvant treatment of NSCLC. Extending to 3–4 cycles instead of 2 cycles of neoadjuvant chemoimmunotherapy may improve the safety of surgery and result in a lower incidence of perioperative morbidities; however, the MPR rate did not increase significantly with more cycles, in this study. CT re-evaluation during treatment and PD-L1 expression at initial diagnosis are potential indicators to guide the choice of the number of therapeutic cycles.

## Data availability statement

The original contributions presented in the study are included in the article/supplementary material. Further inquiries can be directed to the corresponding author.

## Ethics statement

The studies involving humans were approved by the Ethics Committee of Hunan Cancer Hospital (2023032). The studies were conducted in accordance with the local legislation and institutional requirements. The participants provided their written informed consent to participate in this study.

## Author contributions

(I) Conception and design: BZ, QX, XG, RJ, JW, WW, ZW. (II) Administrative support: BZ, QX. (III) Provision of study materials or patients: BZ, DY, XL, WW, QX, XC, JL. (IV) Collection and assembly of data: BZ, QX, ZW, RJ. (V) Data analysis and interpretation: BZ, QX. (VI) Manuscript writing: All authors. (VII) Final approval of manuscript: All authors.
